# Editorial: Associations between Reading and Mathematics: Genetic, Brain Imaging, Cognitive and Educational Perspectives

**DOI:** 10.3389/fpsyg.2017.00600

**Published:** 2017-04-21

**Authors:** Sarit Ashkenazi, Orly Rubinsten, Bert De Smedt

**Affiliations:** ^1^The Seymour Fox School of Education, The Hebrew University of JerusalemJerusalem, Israel; ^2^Edmond J. Safra Brain Research Center for the Study of Learning Disabilities, Department of Learning Disabilities, University of HaifaHaifa, Israel; ^3^Faculty of Psychology and Educational Sciences, University of LeuvenLeuven, Belgium

**Keywords:** reading, mathematics, cognitive, education, neurobiological

Converging evidence demonstrates a strong link between reading and mathematics (LeFevre et al., 2010; Purpura and Ganley, 2014). This research topic aimed to explore the underlying mechanisms of this overlap between reading and mathematics. The empirical studies in this special issue cover three important, although not independent, perspectives including the neurobiological, cognitive, and educational perspectives.

Different aspects of numerical processing are represented in different brain regions. Specifically, the bilateral intraparietal sulcus is assumed to host preverbal innate representations of numerical quantity, whereas the left angular gyrus, in connection with other left-hemispheric perisylvian areas, supports the manipulation of numbers lexically. The first representations are based on spatial processes, and are independent from reading, but, the latter shares similar brain networks and cognitive processes with reading (Dehaene, 1992).

In accordance, mathematics represents heterogeneous cognitive abilities and involves the use of different strategies, which rely differentially on these nonverbal and verbal representations (LeFevre et al., 2010). There are also individual differences in the tendency to use these representations and these may also depend on environmental factors, such as socioeconomic status (SES). For example, Demir-Lira et al. tested neural predictors of math gains (up to 3 years) by examining activations in brain regions related to verbal numerical representations and spatial numerical representations. This association was moderated by SES: Activity in verbal brain regions (left inferior frontal gyrus) predicted math in children with high SES, while activity in spatial brain regions (superior parietal lobe) predicted math in children with low SES.

Atypical development of mathematics and reading tends to co-occur (comorbidity) of mathematics learning disabilities (MD) and reading disabilities (RD) is more common than the prevalence of MD without RD (Von Aster and Shalev, 2007) It has been suggested that these two conditions represent distinct subtypes of MD, i.e., MD-only vs. MD/RD, of which the latter may depend on weaknesses in the verbal code shared with reading (Ashkenazi et al., 2013; Szűcs, 2016). In line with this hypothesis, Slot et al. reported that phonological awareness influenced both math and literacy, and was a shared risk factor for MD, RD and spelling disabilities. On the other hand, Evans and Ullman, suggest that this common mechanism is related to procedural memory and its underlying brain systems.

Multiple cognitive processes are shared between reading and mathematics, including the representation and retrieval of symbolic information, attention, working memory, and cognitive control. This is nicely illustrated by Chu et al. who showed in a 3-year longitudinal study that cognitive skills that are relevant to reading (e.g., phonological awareness) as well as cognitive skills that are specific to mathematics (e.g., sensitivity to relative quantity) predicted preschoolers' mathematics achievement.

To further explore the role of reading in mathematics, Bonifacci et al. compared the abilities of bilingual and monolingual children in numerical and arithmetical tasks with or without verbal components. Interestingly, bilingual children, who had better verbal skills, outperformed monolingual children in numerical tasks with a verbal (e.g., knowledge of digits) but not with nonverbal (e.g., quantity comparison) tasks.

Wei et al. approached this issue of interaction between reading and mathematics by analyzing sex differences. Assuming that males outperform females in spatial ability while females outperform males in verbal abilities, it can be predicted that males should be better in nonverbal mathematical tasks but not in verbal tasks. Wei et al. observed indeed that males outperform females in approximate arithmetic, which require spatial processing, and this difference was explained by gender differences in spatial ability.

Turning to the educational perspective with focus on reading and mathematics in the classroom, Rapoport et al. examined teachers' beliefs and practices about the link between reading and mathematics, by focusing on the role of executive functions. Dowker examined the effect of an individualized numeracy intervention, aiming to further determine whether children with MD-only and children with MD/RD require different interventions. Numeracy, reading comprehension and nonverbal IQ were measured before and after the intervention. Although literacy measures correlated with numeracy, they did not influence children's mathematical progress, or the effectiveness of intervention.

In adults, academic skills are crucial to make decisions in daily life Against this background, Vágvölgyi et al. examined functional illiteracy, defined by the inability to use reading, writing, and calculation skills or his/her own and the community's development. They proposed a new definition and add numerical aspects, in addition to the linguistic aspects, to a definition of functional illiteracy.

To sum, the current research topic adds to unraveling the communalities and differences between reading and mathematics learning and its atypical development. The studies in this research topic point to shared mechanisms (e.g., phonological awareness, procedural learning) as well as mechanisms that are clearly distinct between reading and math (e.g., spatial-numerical processes). Future studies should investigate these mechanisms in more detail at the neural level, by focusing on the overlap in networks between reading and different mathematical tasks. It could also be that these shared and independent mechanisms, and consequently the overlap between reading and mathematics, changes across development (De Smedt et al., 2009). Developmental studies are needed in order to determine how the overlap evolves over developmental time. Finally, there is a need for examining the effects of interventions that focus on factors that are either common or specific to reading and math. If a particular skill is causally related to both reading and mathematics, then interventions focused at this skill should have effects on both reading and mathematics. These studies are also needed from an educational point of view, as effective interventions will help to improve children's mathematics and reading skills.

## Author contributions

All authors listed, have made substantial, direct and intellectual contribution to the work, and approved it for publication.

### Conflict of interest statement

The authors declare that the research was conducted in the absence of any commercial or financial relationships that could be construed as a potential conflict of interest.
